# Student Satisfaction and Physical Health Effects of Online Learning Among Medical and Paramedical Undergraduates in Northern India: A Cross-Sectional Study

**DOI:** 10.7759/cureus.62137

**Published:** 2024-06-11

**Authors:** Aseem Garg, Usharani Rathnam, Varsha Gupta, Sumit Grover, Souvik Manna

**Affiliations:** 1 Internal Medicine, Kalpana Chawla Government Medical College, Karnal, IND; 2 General Surgery, Employees State Insurance Corporation Medical College and Hospital, Alwar, IND; 3 Community Medicine, Government Medical College, Alwar, IND; 4 Ophthalmology, National Cancer Institute, All India Institute of Medical Sciences, New Delhi, New Delhi, IND; 5 Community Medicine, Employees State Insurance Corporation Medical College and Hospital, Alwar, IND

**Keywords:** covid-19, health profession education, student satisfaction, online learning, e-learning

## Abstract

Background: Online modality of pedagogy was introduced in many medical institutes globally, especially during the COVID-19 pandemic. However, these techniques were not evaluated, either with respect to effectiveness or student satisfaction in terms of promoting successful educational outcomes. The current study was conducted to identify determinants of student satisfaction with respect to online learning, in the post-pandemic era.

Methods: A pilot-tested and validated online questionnaire was administered to 370 medical/paramedical students who attended online classes during the pandemic. The students were selected randomly from four different streams of a medical university, representing all the years of study. The questionnaire included Likert-type questions and was divided into two parts: socio-demographic profile and satisfaction with online learning.

Results: The response rate was 81.4%. Overall satisfaction with online learning among students was 35.9% and the areas of most satisfaction for students were user-friendliness of the online portal (65.5%), self-directed responsibilities assigned to pupils (49.9%), faculty accessibility/availability (48.8%) and timely evaluation, test and feedback (47.9%). The areas of most dissatisfaction were lack of personal effect as compared to offline learning (n=71, 23.6%), effect on social life (n=54, 17.9%), and feeling of not belonging to the online session (n=38, 12.6%). Multiple regression analysis demonstrated that having previous exposure to online courses and having a separate room led to more satisfaction, whereas the emergence of health problems led to poor satisfaction with the course. The duration of previous exposure to online courses was not a statistically significant predictor of satisfaction. One or more health problems were reported by 176 (58.5%) of the respondents. Some of the common health problems reported were eye strain (72.8%), headache (56.1%), insomnia (47.2%), stress (35.2%), muscle fatigue (22.6) and tingling sensation (10.6%).

Conclusion: Adopting a combination of online and offline approaches, i.e., blended pedagogy, involving different methods to involve students and their feedback are important to ensure student satisfaction.

## Introduction

The COVID-19 response brought online learning to the forefront and the academic community realized its usefulness and practicality for curriculum delivery globally. The reasons behind this worldwide adoption of online learning were accessibility to knowledge from the home environment, customizable content delivery, content standardization, personalized one-to-one communication, self-pacing, interactivity, and affordability [1]. This has helped medical schools keep their courses running for students even during lockdown to continue the teaching-learning process. The other benefit was the avoidance of gathering in a room, where the respiratory viruses can spread from person to person. However, these modalities have continued even after slackening of the restrictions due to the pandemic, partly due to their effectiveness and ease of delivery.

A systematic review and meta-analysis found that online learning increased undergraduates' cognitive and psychomotor abilities, compared to conventional face-to-face learning [2]. The other metric for evaluating the success of this modality is client satisfaction. Hence, measuring student satisfaction with such online modalities helps us to gauge the success and effectiveness of these modalities. Students’ satisfaction has been defined as the attitude that stems from their experience with the educational services and facilities provided by any college [3]. This attitude is explained by various learning theories: social cognitive theory, the Kirkpatrick model, the interaction equivalency theorem, and social integration theory [4,5]. Social cognitive theory emphasizes the role of environment and behaviour along with reinforcements, expectations, and self-efficacy in the learning process. The Kirkpatrick model, on the other hand, enumerates five stages in the hierarchy of learning from satisfaction (reaction) to meeting student expectations. The interaction theories emphasize the central role of different kinds of interactions: student-teacher, student -content and student-student interaction for effective pedagogy. This was demonstrated by an Indian study on business students, which showed that four predictors, viz. quality of teacher, content design, regular feedback, and expectation of students positively impacted student satisfaction and performance [6]. Therefore, satisfaction can be increased as various interactions are embedded within the learning milieu [7]. This can also be achieved by involving students in formal extracurricular activities in addition to their academic courses. A plethora of evidence shows that academic success is related to student satisfaction in addition to the effectiveness of the learning process, access to various resources, and institutional support [8]. A previous study from Sri Lanka reported that addressing learner motivation and their challenges in an interactive online program improved their satisfaction with the new learning modality [9]. The COVID-19 pandemic helped to bring a paradigm shift in the pedagogical techniques throughout the world, in which online learning became an acceptable method of teaching and learning. This new paradigm in academic circles after the COVID-19 pandemic can be called a ‘new normal’. Online learning has not only augmented the conventional methods but has also replaced them in some areas. This gradual transition from face-to-face learning to increasing dependence on online learning is one of the lessons from the pandemic. Only a few studies, especially from South India have measured student satisfaction among medical and paramedical students in India [10]. In addition, the determinants of satisfaction also need exploration, especially to improve the understanding of this process. Therefore, the current study was conducted to identify factors affecting student satisfaction with online learning among medical and paramedical undergraduate students from North India.

## Materials and methods

A descriptive observational quantitative study was conducted at Pandit Bhagwat Dayal Sharma University of Health Sciences, Rohtak, and its affiliated colleges in Northern India between April and May 2022. The study design was a cross-sectional survey and the study population included all undergraduate students enrolled in medical (Bachelor of Medicine & Bachelor of Surgery, MBBS) and various paramedical courses (e.g., Bachelor of Science (BSc) in Optometry, Pharmacy, Nursing, etc.) at the university. 

The sample size of 300 was calculated based on 41.3% satisfaction reported in the literature; 15% relative error, 95% confidence level, and a non-response rate of 20% [11]. The sampling technique was a stratified random sampling method in which stream (e.g., MBBS, BSc Nursing, BSc Optometry, B Pharm) and year of study were used as strata, and the proportional allocation method was used to obtain the sample size of each stratum. The proportion of students in the various streams was calculated and 80 participants were selected from each of the four years using proportional allocation. The sampling frame consisting of a list of all undergraduates in the medical college was obtained from the academic branch, and separate lists based on year and stream were prepared. From the stratified list, the required number of participants was selected using simple random sampling. The selected students were approached either after their classes or at hostels to participate in the online survey. 

An online questionnaire, along with the participant information sheet (PIS) and consent form, were given to the study participants using computer-assisted personal interview (CAPI). The PIS had details regarding the length of time required for the survey, the place where the personal data will be stored and for how long, name of the investigators, and the purpose of the study.

This was a closed survey, and the contact mode was offline, although data was collected on tablets, mobile phones or computers as per participants' convenience. Questionnaires that were used to measure student perception and satisfaction with online learning were also subjected to validity testing using Rasch analysis. In addition, the usability and technical functionality of the electronic questionnaire were tested before fielding the questionnaire through a pilot study. There was an automatic method for capturing responses from the cloud, and no manual data entry was needed.

The questionnaire was divided into two parts, socio-demographic profile and satisfaction with online learning. There were 25 satisfaction items that were scaled using a five-point Likert, ranging from 1 (strongly agree) to 5 (strongly disagree). The questionnaire was piloted, and Cronbach’s alpha (Person Separation Index in Rasch Analysis) was 0.93 for students’ satisfaction. Some items were rephrased to ensure semantic consistency based on the feedback from respondents in the pilot study. There was no adaptive questioning or skip logic in the tool, and all the questions were made mandatory, to avoid missing data. If any participant missed one or more questions, a completeness check during the submission of the questionnaire highlighted the mandatory items, prompting them to answer the mandatory fields. If the participants were not willing to answer any question, there was provision for a nonresponse option, i.e. such as “not applicable” or “rather not say”, and selection of one response option was enforced. After the submission of the questionnaire, the respondents had the option to view their answers via an email which displayed a summary of the responses and asked the respondents if any corrections were needed. Each completed questionnaire was associated with a unique IP address that was matched to remove duplicates and multiple responses by the same participant. No two entries from the same IP address were analyzed during the study period (April-May 2022) and the most recent entry was kept for analysis.

The study received ethical approval from the Institute Ethics Committee of Kalpana Chawla Government Medical College, Karnal, and conformed to the tenets of the Declaration of Helsinki, 2013. All participants gave online consent, and they were provided with contact information if they wanted to clarify doubts or ask questions. All data were coded to ensure anonymity.

Statistics

RStudio (version 2023.06.1+524 "Mountain Hydrangea" Release) was used for Rasch analysis, while SPSS software, version 26.0 (IBM Corp., Armonk, NY) was used for other statistical analysis. The need for two software was justified because Rasch analysis needs special packages available in RStudio free of cost. The dissatisfaction scores were obtained from RStudio using Rasch analysis and any missing items were analyzed using imputation of its expected value of zero. This forced the correlations to be consistent and the zero residuals dampened the size of factors in the residuals, with little effect on the factor structure.

Descriptive statistics, like mean and standard deviation were used to summarize the scores. Uni-variable analysis was performed first to identify any predictors with p value<0.1, and they were next introduced into the multivariable-adjusted analysis using the backward method. The assumptions of linear regression were satisfied, i.e., linearity, independence, normality of residuals, and homoscedasticity. Assumptions were checked using scatter plots, Q-Q plots, and Kolmogrov-Smirnov tests. Multiple linear regression was run to predict the scores on the satisfaction subscale with socio-demographic variables as independent variables. 

## Results

**Figure 1:**
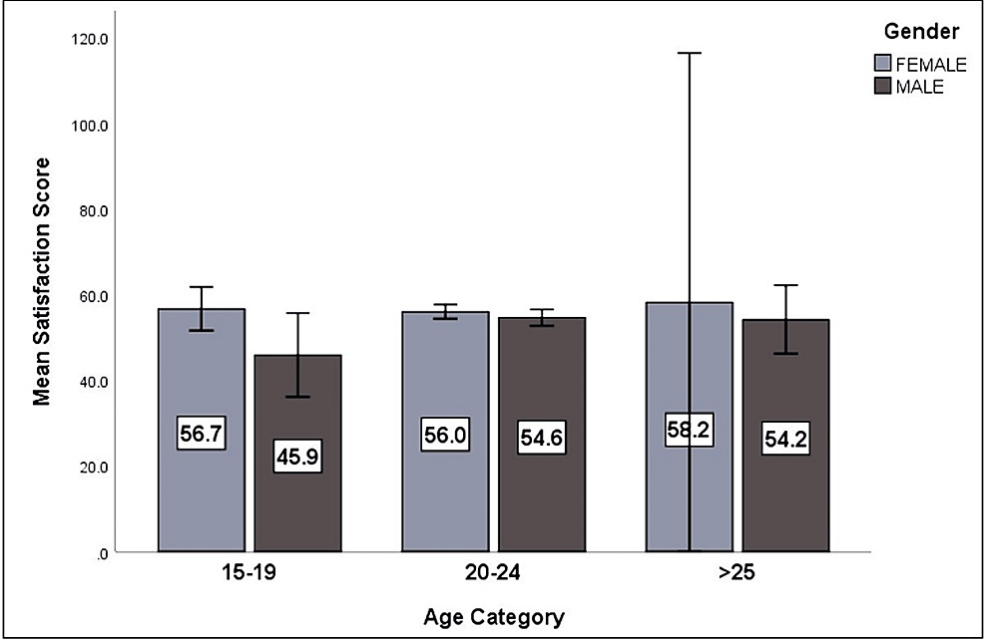
Difference of mean satisfaction (with online learning) scores based on age and gender (N=301)

Three hundred one responses out of 370 were received from students, a response rate of 81.4%, comprising of 169 (56.1%) females and 132 (43.9%) male students. Maximum participants belonged to the age groups 20-24 years (81.4%), followed by 15-19 years (15.6%) and above 25 years (3.0%). Half of the students (48.2%, n = 145) had no previous experience with online learning. Gender and age did not have any influence on the scores, as evident from the similar mean scores in both genders in all age groups (Figure 1).

Overall satisfaction with online learning among students was 35.9% and the areas of most satisfaction for students were user-friendliness of the online portal (65.5%), self-directed responsibilities assigned to pupils (49.9%), faculty accessibility/availability (48.8%) and timely evaluation, test and feedback (47.9%) (Table 1).

**Table 1:** Response frequency of the 25-item questionnaire The 25-item questionnaire was used for the assessment of satisfaction with online learning among medical and paramedical students (N=301). *Reverse Coded Item, F2F: face to face

Sr.	Items	Strongly Agree (%)	Agree (%)	Neutral (%)	Disagree (%)	Strongly Disagree (%)	Not applicable/rather not say (%)
1	There was clear communication of class assignments	12 (4.0)	96 (31.9)	135 (44.9)	31 (10.3)	15 (5.0)	12 (4.0)
2	Evaluation, test and feedback were given on time	11 (3.7)	133 (44.2)	98 (32.6)	32 (10.6)	16 (5.3)	11 (3.7)
3	I felt part of the class and belonged to the online session	11 (3.7)	90 (29.9)	138 (45.8)	24 (8.0)	23 (7.6)	15 (5.0)
4	Satisfied with faculty accessibility/availability	17 (5.6)	130 (43.2)	98 (32.6)	28 (9.3)	15 (5.0)	13 (4.3)
5	Satisfied with online discussion forums	11 (3.7)	101 (33.6)	118 (39.2)	45 (15.0)	13 (4.3)	13 (4.3)
6	Satisfied with online communication including email & announcements	17 (5.6)	123 (40.9)	98 (32.6)	39 (13.0)	12 (4.0)	12 (4.0)
7	Online learning management system is user friendly	68 (22.6)	126 (41.9)	78 (25.9)	10 (3.3)	7 (2.3)	12 (4.0)
8	Satisfied with the download duration of learning resources.	16 (5.3)	105 (34.9)	117 (38.9)	38 (12.6)	10 (3.3)	15 (5.0)
9	Satisfied with the number of online sessions	10 (3.3)	118 (39.2)	109 (36.2)	36 (12.0)	15 (5.0)	13 (4.3)
10	Online courses offered flexible timings	20 (6.6)	115 (38.2)	91 (30.2)	41 (13.6)	19 (6.3)	15 (5.0)
11	Satisfied with the self-directed responsibilities assigned to me	18 (6.0)	132 (43.9)	108 (35.9)	16 (5.3)	12 (4.0)	15 (5.0)
12	Enjoyed working on projects during online courses	8 (2.7)	88 (29.2)	123 (40.9)	46 (15.3)	20 (6.6)	16 (5.3)
13	Satisfied with quality of interaction between me, faculty and peers	14 (4.7)	79 (26.2)	128 (42.5)	46 (15.3)	17 (5.6)	17 (5.6)
14	Satisfied with collaborative activities during online learning	10 (3.3)	86 (28.6)	138 (45.8)	32 (10.6)	19 (6.3)	16 (5.3)
15	Can relate my level of understanding to the other students	4 (1.3)	113 (37.5)	118 (39.2)	34 (11.3)	16 (5.3)	16 (5.3)
16	Comfortable with participating in online sessions	9 (3.0)	106 (35.2)	112 (37.2)	42 (14.0)	17 (5.6)	15 (5.0)
17	Satisfied with level of required effort in online courses	13 (4.3)	105 (34.9)	113 (37.5)	41 (13.6)	13 (4.3)	16 (5.3)
18	Satisfied with my performance in online course	9 (3.0)	95 (31.6)	107 (35.5)	56 (18.6)	19 (6.3)	15 (5.0)
19	Satisfied with my final grade	11 (3.7)	95 (31.6)	122 (40.5)	39 (13.0)	15 (5.0)	19 (6.3)
20	Able to apply what I learned in this online course	10 (3.3)	96 (31.9)	114 (37.9)	53 (17.6)	15 (5.0)	13 (4.3)
21	Will recommend this online learning experience to others	7 (2.3)	80 (26.6)	100 (33.2)	62 (20.6)	38 (12.6)	14 (4.7)
22	More satisfied with online learning compared to F2F session	11 (3.7)	51 (16.9)	77 (25.6)	91 (30.2)	58 (19.3)	13 (4.3)
23	Satisfaction encourages me to register in other available online courses.	6 (2.0)	71 (23.6)	120 (39.9)	61 (20.3)	29 (9.6)	14 (4.7)
24	Online classes have affected my social life in colleges.*	11 (3.7)	27 (9.0)	108 (35.9)	101 (33.6)	39 (13.0)	15 (5.0)
25	Overall, I am satisfied with the course	15 (5.0)	93 (30.9)	120 (39.9)	37 (12.3)	21 (7.0)	15 (5.0)

The areas of most dissatisfaction reported by students were lack of personal interaction as compared to face-to-face teaching (n=71, 23.6%), effect on social life (n=54, 17.9%) and feeling of not belonging to the online session (n=38, 12.6%). The other main challenges reported by students were inability to work on projects (n=36, 11.9%), and other collaborative activities (n=35, 11.6%). Moreover, 17.3% (n=52) were not willing to recommend the online learning experience to others and 14.3% (n=43) were discouraged to enroll in other online courses due to their poor satisfaction (Table 1).

**Figure 2:**
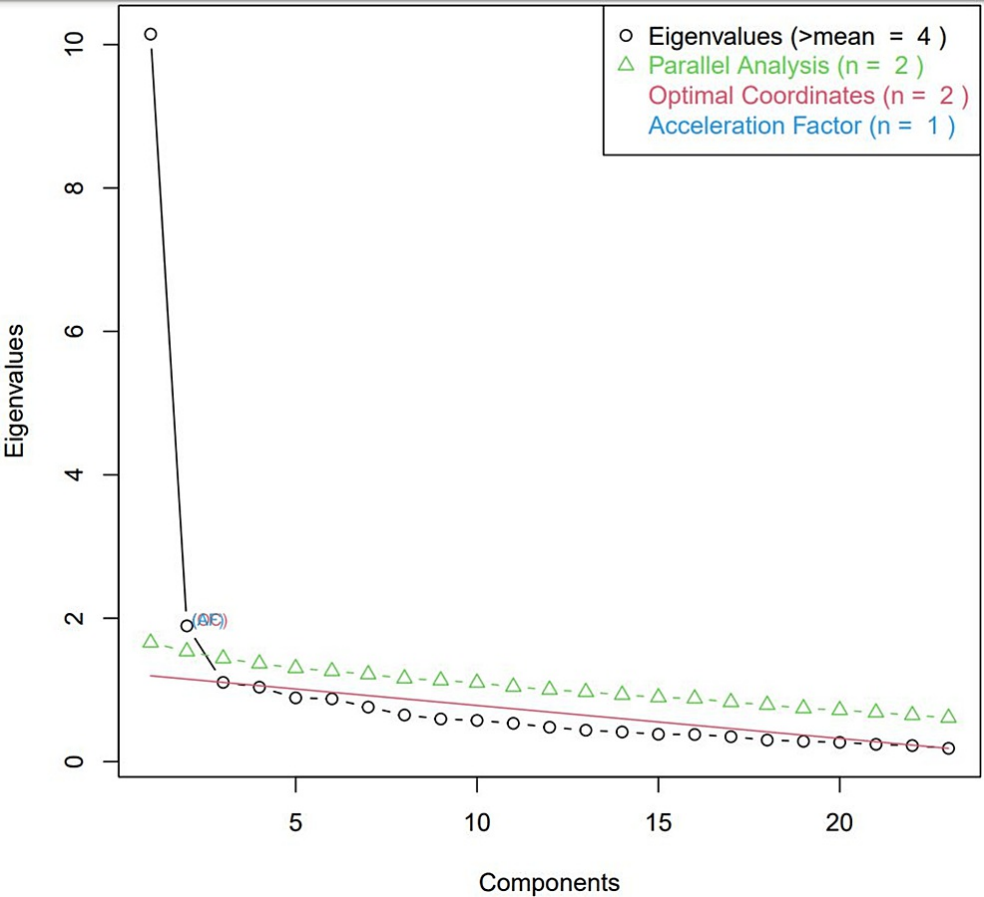
Scree plot after factor analysis of the satisfaction items Eigenvalues are a measure of the amount of variance accounted for by a factor, and so they are useful in determining the number of factors that we need to extract. In this scree plot, authors plotted the eigenvalues for all of the factors, and then looked for the sharp drop.

Rasch analysis was done to check the validity and reliability of the tool. It is a psychometric technique that helps researchers improve the precision of questionnaires, monitor its quality, and calculate continuous performance scores from ordinal data [12]. The reasons for using Rasch analysis included checking for instrument unidimensionality, differential item functioning, rating categories, item hierarchy, and finding redundant items [13]. The three assumptions of the Rasch model, item independence, unidimensionality, and monotonicity, were checked by fitting the 25 items into the Rasch model. The item dependence plot showed that multiple items were correlated with each other and needed to be merged to generate the cumulative score. The items 1,2,4,5 and 6 were all dependent on each other as the correlation coefficients of the residuals were above 0.2. All these items related to communication and feedback during online classes and needed to be merged. Similarly, items 7, 8 related to technical aspects of the hardware, 12, 13 related to collaborative activities, and 14-16 related to participatory activities. Items 17-22 related to the performance and assessment after the course were merged. The unidimensionality of the Rasch model was checked from the parallel analysis scree plot, which demonstrated one major latent construct with Eigen value=10 (Figure 2).

Finally, the assumption of monotonicity was checked from the person item map, which showed that all the items followed stochastic thresholds. The Rasch analysis gave a metric score for each participant, which was based on the satisfaction of the participant and the item difficulty of the individual questions. This score was an interval score ranging from 0-100 and could be used as a continuous variable for comparisons and regression.

The student’s t-test was done to compare mean student satisfaction based on previous experience in online learning. Previous experience in online learning led to more satisfaction (53.9 ± 11.7) as compared to no experience (56.4 ± 9.7), and the difference was statistically significant (t=-1.93, p=.047). Gender does not affect student satisfaction, as there was no statistically significant difference between the satisfaction scores of males (53.8 ± 10.8) and female students (56.2 ± 10.6), t=-1.8, p=.067.

One or more health problems were reported by 176 (58.5%) of the respondents. Some of the common health problems reported were eye strain (72.8%), headache (56.1%), insomnia (47.2%), stress (35.2%), muscle fatigue (22.6) and tingling sensation (10.6%).

Adjusted regression analysis was performed to identify the predictors of dissatisfaction with online learning, which demonstrated that having previous exposure to online courses and having a separate room led to more satisfaction, whereas emergence of health problems led to poor satisfaction with the course. The duration of exposure was not a significant predictor of exposure (Table 2).

**Table 2:** Multiple regression showing the predictors of dissatisfaction with online courses (N=301) *SE: Standard error

Predictors	Unstandardized Beta (95% CI)	SE	Standardized Beta	t	Sig.
(Constant)	52.348 (46.838, 57.858)	2.811		18.622	.000
Gender	2.391 (-0.139, 4.921)	1.291	0.111	1.851	.065
Previous Exposure	2.765 (0.299, 5.231)	1.258	0.129	2.198	.029*
Separate Room	3.525 (0.975, 6.075)	1.301	0.162	2.709	.007*
Health Issues	-3.784 (-1.226, -6.342)	1.305	-0.172	-2.901	.004*
2-4 hrs	-2.757 (0.82, -6.334)	1.825	-0.127	-1.511	.132
4-6 hrs	-3.586 (0.771, -7.943)	2.223	-0.126	-1.613	.108
> 6 hrs	2.309 (-2.749, 7.368)	2.581	0.065	0.894	.372

## Discussion

Competency-based medical education and micro-teaching techniques have been incorporated into medical education throughout the world for improving student satisfaction and skill upgradation. The present study found that more than one-third of participants (35.9%) were satisfied with the user-friendliness of the online portal (65.5%), self-directed responsibilities assigned to pupils (49.9%), faculty accessibility/availability (48.8%) and timely evaluation, test and feedback (47.9%). Other areas of satisfaction were clear communication of class assignments (35.9%), final grades obtained (35.2%), application of knowledge (35.2%) and performance (34.6%). Client satisfaction with online learning was associated with several factors, such as previous exposure, having a separate room for study, and having good health. The current study on student satisfaction was based on a tool validated on the same population; earlier studies measured student satisfaction with tools validated in other countries [14].

Students reported satisfaction with the user-friendliness of the online portal, self-directed learning, faculty accessibility/availability and timely evaluation and feedback. A plethora of evidence, especially those based on the interaction equivalence theorem have reported that both the quantity and quality of student interactions are highly correlated with their satisfaction in the learning milieu [15]. The areas in which students were most dissatisfied were lack of personal interaction in online mode as compared to face-to-face teaching, effect on social life, and feeling of not belonging to the online session or not feeling ownership. Previous studies have reported that most students were dissatisfied because of issues related to workload, student engagement, and time spent preparing for exams [16]. The present study corroborates these earlier studies, and students reported a lack of participation and a didactive nature of course as a factor leading to dissatisfaction. The interaction equivalence theorem can be amply justified based on the findings of this study as well. Another interesting finding was that the proportion of students satisfied with online learning (35.9%) was higher than those dissatisfied (15.3%), whereas 45% of the students were neutral. This corroborates previous studies that have shown that most students were either neutral or satisfied with online learning [17].

The common health problems due to prolonged use of digital devices reported in the current study were eye strain (72.8%), headache (56.1%), insomnia (47.2%), stress (35.2%), muscle fatigue (22.6) and tingling sensation (10.6%). Exposure to ultra-violet light emitted from digital devices can lead to a plethora of health problems, e.g., an increase in the amount of deoxyribonucleic acid (DNA) damage, cell injury, tissue death, vision problems, skin barrier damage, and photoaging [18]. Nearly half of the participants exposed to blue light reported change in their sleeping pattern; a finding well documented by previous studies [19,20]. Ocular symptoms like eye strain are common symptoms associated with myopia progression. Previous studies have shown an association between blue light exposure and myopia such that every 1-hour increase in blue light exposure daily is associated with 1.26 times (odds ratio (OR): 1.26, 95% CI: 1.21-1.31, p < 0.001) higher risks of myopia progression [21].

The current study showed that students’ satisfaction was associated with factors like having previous exposure to online courses and having a separate room, whereas the emergence of health problems led to poor satisfaction with the course. The duration of exposure was not a significant predictor of exposure. Previous studies have explored the role of technology-related factors, like technical support in the form of helplines, chatbots, customer service agents, user-friendliness of the technological infrastructure, etc [22]. Other studies have reported that students now expect round-the-clock availability of the faculty to respond to their late queries, emails, calls, and doubts on online discussions [23].

A synthesis of over 100 meta-analyses has reported that micro-teaching and feedback were among the ten most important aspects having a correlation with student achievement and satisfaction [24]. The feedback can be in the form of surveys, questionnaires, post-test assessments, or simple verbal comments on the teaching style. Effective feedback should be timely to enhance students’ learning and help them monitor their progress [25]. Similarly, in the techniques of micro-teaching, recorded sessions of the class are being shown to peers, students and experts for their comments and feedback for improvement of the teacher [26]. Informal or verbal feedback also has the potential to enhance communication among peers and faculty as it offers ways to maintain or improve teaching skills [27].

The current study has implications in the quality improvement of online learning in India. The newer competency-based medical education (CBME) modules also stress the need of improving and developing newer methods of teaching and assessment for overall skills training of the medical undergraduates [28-29]. The study recommends a mixture of offline and online approaches, i.e. blended techniques, incorporating the strengths and opportunities in different applications to engage students in online learning [11].

One of the strengths of the study is that it documented the factors associated with student satisfaction, and those leading to dissatisfaction. Further, the questionnaire used was tested using the Rasch model, which gave a metric score of dissatisfaction, used for the regression analysis. The limitation of this study is the use of a self-assessment questionnaire, which can be influenced by social desirability and recall biases. Also, a small sample size limits the generalizability of the findings and leads to reduced external validity.

## Conclusions

Online learning has been a game-changer during COVID-19 and its pragmatic application for pedagogy has huge potential. However, with proper interaction between students and teachers and flexibility of curriculum, online learning can achieve its stated goals in a more effective manner. Other challenges among students were the emergence of health problems, lack of engagement and lack of participation. Only a multipronged strategy that maximises the benefits to students while minimizing the disadvantages can lead to an overall improvement in student satisfaction with online learning.
